# Effects of lipopolysaccharide-induced inflammation on expression of growth-associated genes by corticospinal neurons

**DOI:** 10.1186/1471-2202-7-8

**Published:** 2006-01-24

**Authors:** MK Hossain-Ibrahim, K Rezajooi, JK MacNally, MRJ Mason, AR Lieberman, PN Anderson

**Affiliations:** 1 Department of Anatomy and Developmental Biology, University College London, Gower Street, London WC1E 6BT, UK

## Abstract

**Background:**

Inflammation around cell bodies of primary sensory neurons and retinal ganglion cells enhances expression of neuronal growth-associated genes and stimulates axonal regeneration. We have asked if inflammation would have similar effects on corticospinal neurons, which normally show little response to spinal cord injury. Lipopolysaccharide (LPS) was applied onto the pial surface of the motor cortex of adult rats with or without concomitant injury of the corticospinal tract at C4. Inflammation around corticospinal tract cell bodies in the motor cortex was assessed by immunohistochemistry for OX42 (a microglia and macrophage marker). Expression of growth-associated genes c-jun, ATF3, SCG10 and GAP-43 was investigated by immunohistochemistry or in situ hybridisation.

**Results:**

Application of LPS induced a gradient of inflammation through the full depth of the motor cortex and promoted c-Jun and SCG10 expression for up to 2 weeks, and GAP-43 upregulation for 3 days by many corticospinal neurons, but had very limited effects on neuronal ATF3 expression. However, many glial cells in the subcortical white matter upregulated ATF3. LPS did not promote sprouting of anterogradely labelled corticospinal axons, which did not grow into or beyond a cervical lesion site.

**Conclusion:**

Inflammation produced by topical application of LPS promoted increased expression of some growth-associated genes in the cell bodies of corticospinal neurons, but was insufficient to promote regeneration of the corticospinal tract.

## Background

Evidence from studies on dorsal root ganglion (DRG) neurons and retinal ganglion cells (RGCs) suggests that the induction of an inflammatory response around the cell bodies of axotomised neurons enhances the regeneration of their axons. Injection of corynebacterium into DRG prior to dorsal root injury produces a fourfold increase in the number of regenerating axons [1]. Similarly, injection of zymosan into the vitreous body of the eye induces extensive but transient regeneration of RGC axons in the crushed optic nerve [2]. Lens injury has similar effects on RGC axonal regeneration, probably because it stimulates the accumulation of macrophages in the retina [2]. Furthermore, adult RGCs grown in media conditioned by activated macrophages also display enhanced neurite growth [3].

Against this background we have investigated the possibility that LPS-induced inflammation around the cell bodies of corticospinal tract neurons would enhance their expression of growth-associated genes (c-jun, ATF3, SCG10 and GAP-43) and promote regeneration of their axons following spinal cord injury. It is of particular importance and interest to investigate these questions in relation to corticospinal neurons because their axons constitute the major descending motor pathway, and because they display very poor regenerative responses to injury. Thus corticospinal neurons do not regenerate axons to any significant extent along peripheral nerve grafts inserted into the spinal cord or brainstem [4-6] and display upregulation of growth-associated genes only after intracortical lesion of their axons and not after spinal injury [7,8].

## Results

### Assessing LPS-induced inflammation in cortex

Coronal sections through the motor cortex to which LPS had been applied were immunostained with OX42, an antibody that recognizes the type 3 complement receptor (CR3) in mononuclear phagocytes, which is upregulated by activated microglia and macrophages [9]. In unoperated brains and on the side opposite to LPS application, and in sham-operated animals (dura opened; no LPS applied +/- Gelfoam), highly ramified (presumably resting) microglia were present throughout the cortex. In brains in which the dura had been opened contralateral to the side of LPS application, there was some accumulation of rounded OX42-positive cells at the pial surface in the first 7 days after treatment but deeper layers contained ramified microglia (Figs. 2a, c, e). In LPS-treated cortex, microglial morphology was altered from the ramified form typical of the quiescent state, to a rounded amoeboid shape, with thicker proximal processes and loss of distal ramification (Figs 2b, d). At 3 days after LPS application microglia numbers in layer V were increased, with a mean of 177 cells per unit area (136,000 μm^2^), 91.4% of which were activated, compared to a mean of 69 cells in the contralateral cortex – of which only 0.5% were activated (Table 1). In the animals in which Fluorogold was used to retrogradely label corticospinal tract (CST) neurons, activated OX42-positive microglia were closely associated with the cell bodies of identified CST neurons in layer V (Fig. 3). However, the inflammatory response to LPS was variable. In most animals, microglial activation was seen in all layers of the cortex beneath the site of LPS application, and extending up to 2 mm on either side, for up to one week after application. In a few animals microglial activation was only evident in the outer two thirds of the cortex. Inflammation around layer V pyramidal neurons was always apparent beneath the site of LPS application. Two weeks after application, rounded, amoeboid microglia were rare and microglia with a ramified morphology predominated in both the ipsilateral and contralateral cortex (Figs 2e, f). There were 75.3 microglia per unit area in control cortex, 0.4% of which were activated, compared to 79.3 microglia per unit area, of which 2.9% were activated, in LPS-treated cortex. At one month, the ipsilateral and contralateral cortex appeared identical in all animals, with only ramified microglial cells apparent. Thus, 500 μg of LPS was sufficient to produce inflammation around layer V pyramidal neurons in all animals by 24 hours, which was maximal at 3 days and had subsided by two weeks after application.

**Figure 1:**
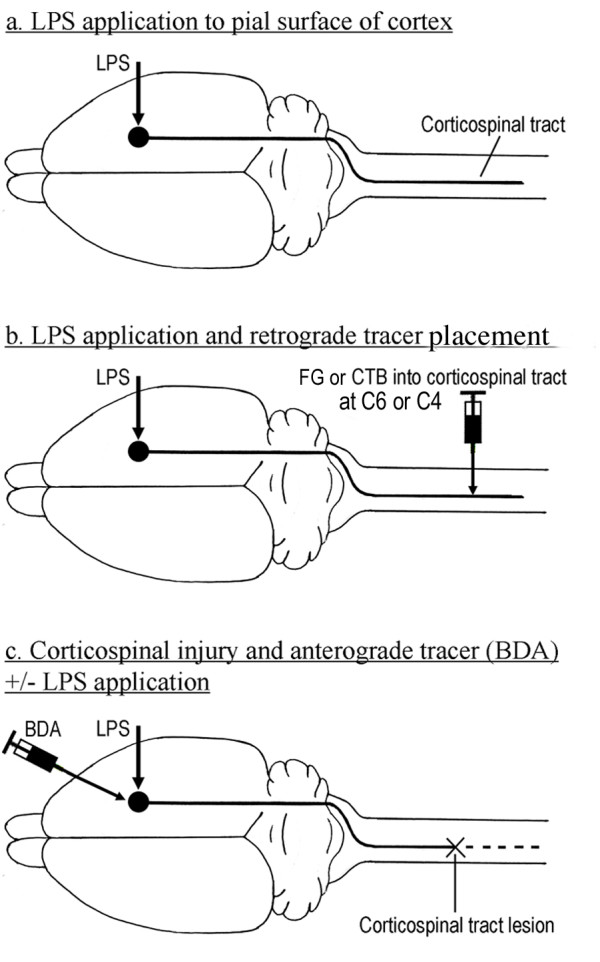
**Schematic diagrams showing experimental design and procedures**. **a**. Unilateral application of lipopolysaccharide (LPS) to the pial surface of motor cortex through a cranial burrhole with sham operation on the contralateral side, to investigate the inflammatory response and expression of growth-associated proteins. **b**. Unilateral LPS application to the pial surface and injection of Cholera toxin B or placement of Fluorogold (FG) (retrograde tracers) into the contralateral corticospinal tract (CST) at C4 or C6 respectively, to identify CST neurons displaying changes in growth-associated protein expression. **c**. Application to the pial surface of LPS with injection of biotinylated dextran amine (BDA; anterograde tracer) into motor cortex and transection of contralateral CST. LPS application omitted in control animals.

**Figure 2:**
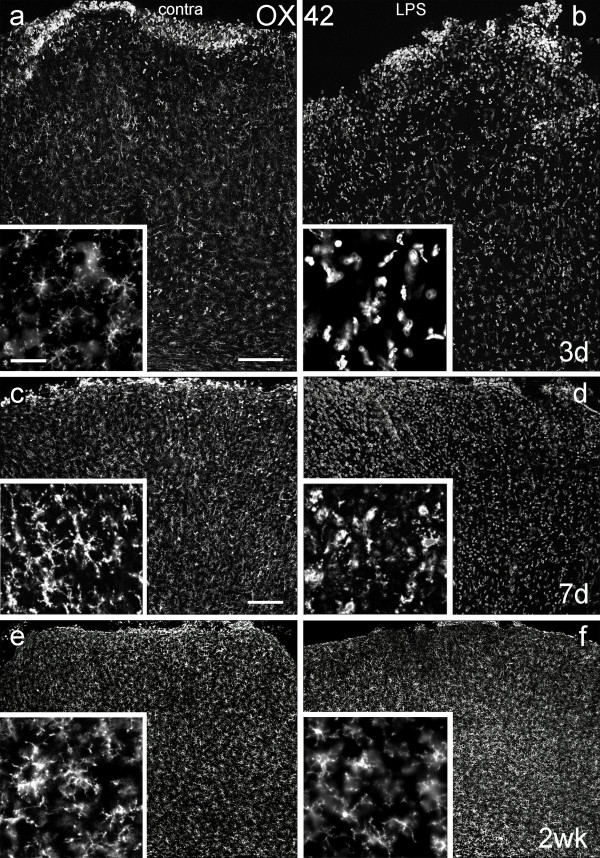
**Microglial responses to LPS application**. Coronal sections of motor cortex, immunoreacted with OX42 antibody to visualise microglia/macrophages 3 days (Figs 2a, b), 7 days (Figs 2c, d) and 2 weeks (Figs 2e, f) after unilateral application of LPS to the pial surface (Figs 2b, d, f) or sham operations on the contralateral (contra), control side (Figs 2a, c, e). Here and in all other figures, the pial surface is at the top and all sections are photographed immediately below the craniotomy, with the same exposure for all pairs of images taken at each survival time. Microglia from layer V are illustrated at higher magnification in the insets. Note that microglia are present throughout the full depth of cortex at all time points and are ramified in the control but are rounded and amoeboid and more numerous in the LPS-treated cortical tissue 3 days and 7 days after LPS application. The numerous immunoreactive cells at the pial surface on both sides of the brain (Figs 2a, b) are likely to be macrophages of haematogenous origin induced by local damage due to craniotomy. Note the reduction in number of such cells at 7 days and that very few remain at two weeks. Scale bar in Fig. 2a = 200 μm and also applies to Fig. 2b; scale bar in Fig. 2c = 200 μm and also applies to Figs 2d – f (Figs 2a and b are of greater magnification than Figs. 2c – f); scale bar in the inset to Fig. 2a = 50 μm and applies to all insets.

**Table 1:** Number and morphology of cortical layer V microglia after sham-operation or LPS application

		Sham-operated		LPS-treated	
		
		Ramified microglia	Activated microglia	Ramified microglia	Activated microglia
3 days after LPS	Mean number	69	0.3	15.3	162
	% of total	99.5	0.5	8.6	91.4
14 days after LPS	Mean number	75.3	0.3	77	2.3
	% of total	99.6	0.4	97.1	2.9
1 month after LPS	Mean number	67	0	72	0
	% of total	100	0	100	0

*Note*: All microglia within an area of 400 μm × 340 μm (136,000 μm^2^) defined by a counting frame superimposed over a digitised image of layer V were counted and identified as either ramified (quiescent) or activated. Numbers represent means of 3 counts; % indicates relative proportions of ramified and activated microglia.

**Table 2:** Expression of cortical growth-associated genes in unoperated, sham-operated and LPS-treated animals

**Group**	**c-Jun **expression cortical layers	**ATF3 **expression cortical layers	**SCG10 **expression cortical layers	**GAP-43 **expression cortical layers
Unoperated animals	-/+	-	-/+	+
	II, III		V	II – VI
Control animals (dura opened)	+	+	-/+	+
	II, III, V	I, II	V	II – VI
Cortex contralateral to LPS application	+	+	-/+	+
	II, III, V	I, II	V	II – VI
LPS-treated cortex	+++	++	+	++
	II, III, V^1^	I, II^2^, V^4 ^& white matter glia^2^	V^1^	II – VI^3^

*Note*: Superscript numbers refer to duration of expression of the growth-associated gene after LPS application: ^1 ^for up to 2 weeks; ^2 ^for up to 7 days; ^3 ^for up to 3 days; ^4 ^for up to 24 hours

**Figure 3:**
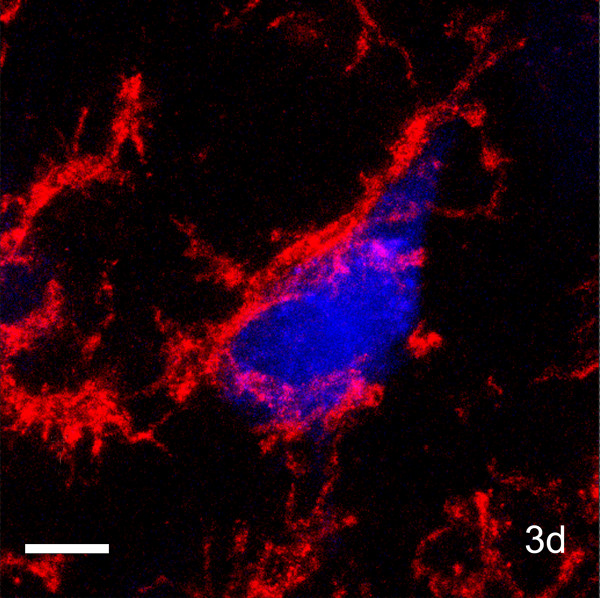
**Microglial response around identified CST neuron after LPS application**. Coronal section of OX42-immunoreacted motor cortex (layer V) 3 days after LPS administration and simultaneous application of Fluorogold to a lesion of contralateral cord at C6. Note the very close association between the microglial cell (red) and the retrogradely-labelled (blue) CST neuronal cell body. Scale bar = 10 μm.

**Figure 4:**
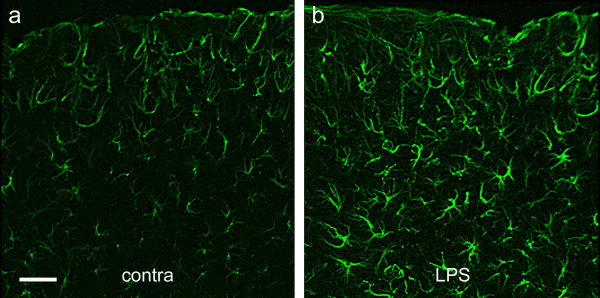
**Astrocytic response to LPS application**. Coronal sections of GFAP-immunostained motor cortex below the area of LPS application (Fig. 4b) or sham operation (Fig. 4a) on the contralateral (contra), control side, 3 days after application of LPS. Note that astrocytes are present throughout the full depth of cortex and are more brightly fluorescent in the LPS-treated cortex, with thicker processes than in the control cortex. Scale bar in Fig. 4a = 200 μm and also applies to Fig. 4b.

From 3 days to 2 weeks after the application of LPS, GFAP-positive astrocytes in the LPS-treated cortex showed hypertrophy of their processes and GFAP immunofluorescence near the inflamed pial surface was more intense (Fig. 4b) than in the contralateral cortex, (Fig. 4a) or in unoperated controls. Astrogliosis was less marked in deeper layers of cortex.

### Expression of growth-associated genes by corticospinal tract neurons

**Figure 5:**
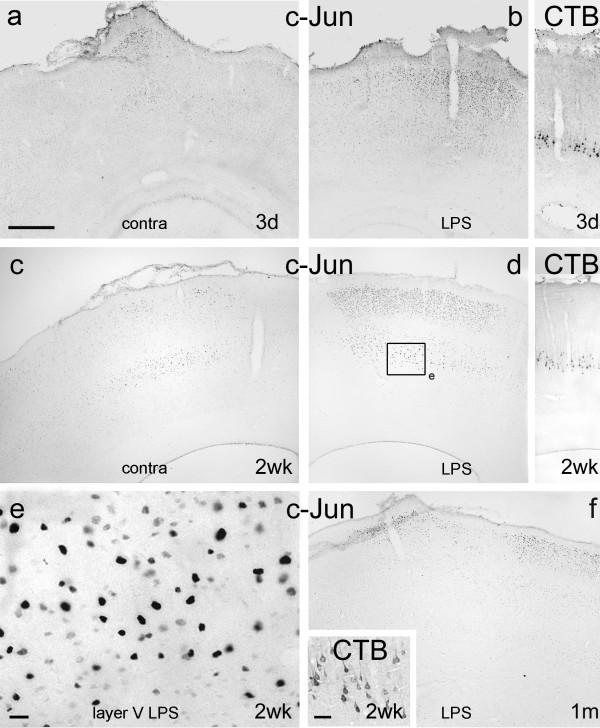
**Expression of c-Jun after LPS application**. Coronal sections of motor cortex 3 days (Figs 5a, b), 2 weeks (Figs 5c – e) and 1 month (Fig. 5f) after unilateral application of LPS, or sham operation (Figs 5a, c), immunoreacted for c-Jun or CTB (insets to Figs 5b, d and f). Note that, at 3 days c-Jun is detectable in layers II, III and V at low levels immediately below the craniotomy on the control side, but is almost undetectable more medially and laterally, and that c-Jun immunoreactivity is much stronger on the treated side, predominantly in layers II, III and V, immediately below the site of LPS application. Note also the marked increase in c-Jun immunoreactivity in layers II, III and V immediately below the burr hole and site of LPS application in Fig. 5d compared to the corresponding contralateral cortex in Fig. 5c. The framed area of layer V in Fig. 5d is enlarged in Fig. 5e to show details of immunostained nuclei. The insets to Fig. 5b and d are taken from the section immediately serial to the ones shown in Figs 5b and d and demonstrates that retrogradely labelled CST neurons occupy the same area (in layer V) as neurons displaying upregulation of c-Jun expression. Some of the retrogradely labelled cells in Fig. 5d are shown at greater magnification in the inset to Fig. 5f. Note also that at one month, c-Jun immunoreactivity in layers II and III of the experimental side still involves areas medial and lateral to the site of LPS application with almost no c-Jun detectable in layer V (c-Jun immunoreactivity in the contralateral cortex is weak and largely confined to layers II and III: not shown). Scale bar = 500 μm and applies to Figs 5a – d and f); bar in Fig. 5e = 20 μm; bar in Fig. 5f inset = 50 μm.

Injection of CTB into the spinal cord at C4 invariably labelled layer V pyramidal cell bodies in the contralateral motor cortex. Sections immediately adjacent to those reacted for CTB were reacted for c-Jun, ATF3 or SCG10 (e.g. Figs 5b, d). Other sections were reacted for both CTB and a growth-associated protein, allowing identification of the protein and CST neurons in the same section (Fig. 8).

#### Control cortex

Making a burr hole in the skull and opening the dura had some effect on c-Jun and ATF3 expression for up to 3 days. In unoperated cortex, c-Jun was weakly expressed in cells in layers II, III and V. There was a small increase in c-Jun expression by cells in layers I, II, III and V (mostly or entirely neurons) directly underneath the burr hole even without LPS application (Fig. 5a). In unoperated cortex there was no ATF3 expression whereas in sham-operated controls, ATF3 was expressed by very small numbers of layer I cells directly underneath the burr hole (<5 per section). These ATF3-expressing cells had abnormal nuclei and were possibly apoptotic but could not be unequivocally identified. Expression of SCG10 protein and GAP-43 mRNA were not affected by opening of the dura. SCG10 was weakly expressed, in layer V neurons only, in the cortex contralateral to LPS application, and in sham-operated and unoperated animals. GAP-43 mRNA was present in neurons in all layers of the cortex, but most strongly in layers IV and V, contralateral to LPS application, in sham-operated and unoperated animals, confirming our previous findings [8]. The presence or absence of Gelfoam made no difference to the expression of growth-associated genes in the cerebral cortex.

#### LPS-treated cortex

##### C-Jun

From 24 hours to two weeks after LPS application c-Jun was markedly upregulated in most neurons of layers II, III and V of the motor cortex (Figs 5b, d). Retrograde labelling with CTB (Fig. 5 insets) and retrograde labelling combined with immunohistochemistry for c-Jun (Fig. 8) showed that CST neurons in layer V were among cells upregulating c-Jun in response to LPS application. By 1 month after application (Fig. 5f) there were no detectable differences between treated and control cortex.

##### ATF3

**Figure 6:**
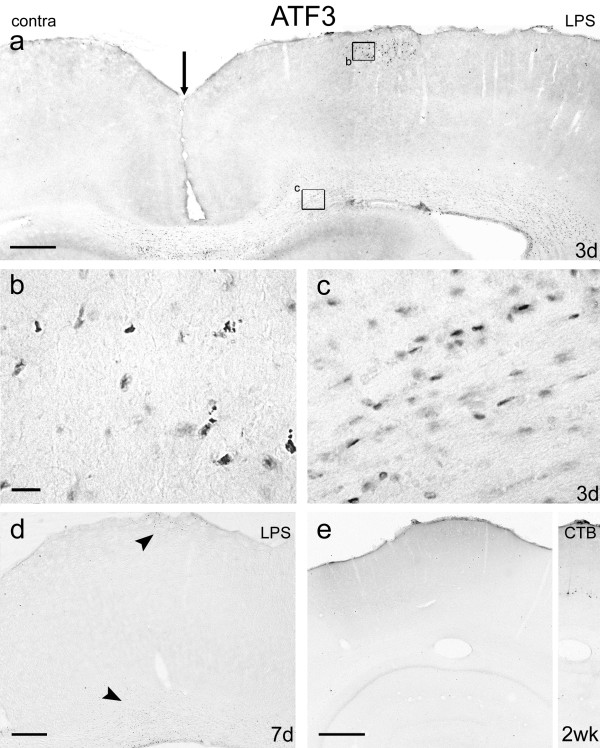
**Expression of ATF3 after LPS application**. Coronal sections of motor cortex immunoreacted for ATF3, 3 days (Figs 6a, b, c), 7 days (Fig. 6d) and 2 weeks (Fig. 6e) after LPS application. Fig. 6a shows both the experimental and the medial part of the control cortex (midline at the vertical arrow) and demonstrates ATF3 immunoreactivity directly under the area of the burr hole and in the subcortical white matter on the experimental side. The superficial upregulation is localised, but immunoreactive cells in the white matter extend for 2 mm laterally and 400 μm across the midline. There is no ATF3 immunoreactivity in cortical neurons located in layers III to VI or in the contralateral (control) cortex. Fig. 6b is enlarged from the boxed area of cortex directly under the site of LPS application in Fig. 6a. Note the irregular shape of ATF3-positive nuclei, suggesting possible damage or apoptosis. Fig. 6c is enlarged from the boxed area of white matter in Fig. 6a. ATF3-positive nuclei are seen arranged in a linear fashion, suggesting that they are white matter glial cells. Fig. 6d, 7 days after LPS application shows reduced ATF3 immunoreactivity directly under the LPS application site (top arrow) and in the subcortical white matter (bottom arrow). Fig. 6e shows no ATF3 immunoreactivity 2 weeks after LPS application. The inset is from the immediately serial section, directly beneath the LPS application site and demonstrates CST neurons retrogradely labelled with CTB. No ATF3 immunoreactivity was seen in the area of cortex where the CTB-labelled CST neurons were located. Scale bar in Figs 6a and e = 500 μm; bar in Fig. 6b = 50 μm and also applies to Fig. 6c; bar in Fig. 6d = 200 μm.

By 24 hours after application of LPS, ATF3 was upregulated in a very few neurons, confined to the region under the burr hole, and in numerous glia in the subcortical white matter. As in the sham-operated animals, ATF3-positive nuclei in layer I were often abnormally shaped. ATF3-positive neurons in layers II to VI were extremely sparse (<8 per section on the treated side; none on the contralateral side), but retained a normal morphology. ATF3 was upregulated in layer V pyramidal neurons but only in relatively small numbers of such cells. Expression of ATF3 in subcortical white matter glia was much more extensive than the neuronal expression; ATF3-positive nuclei were present up to 400 μm into the contralateral hemisphere and for 2 mm lateral to the area of LPS application (not illustrated). At 3 days, neurons expressing ATF3 were almost entirely confined to layers I and II under the burr hole and to glia in subcortical white matter (Figs 6a, b, c). This upregulation was variable and appeared to correlate with the level of inflammation. ATF3 expression was weaker at 7 days (Fig. 6d) and had disappeared by 2 weeks (Fig. 6e). Thus, ATF3 immunoreactivity was seen in only a few layer V pyramidal neurons in animals with a maximal (i.e. full cortical thickness) inflammatory response and only at 24 hours after application.

##### SCG10

**Figure 7:**
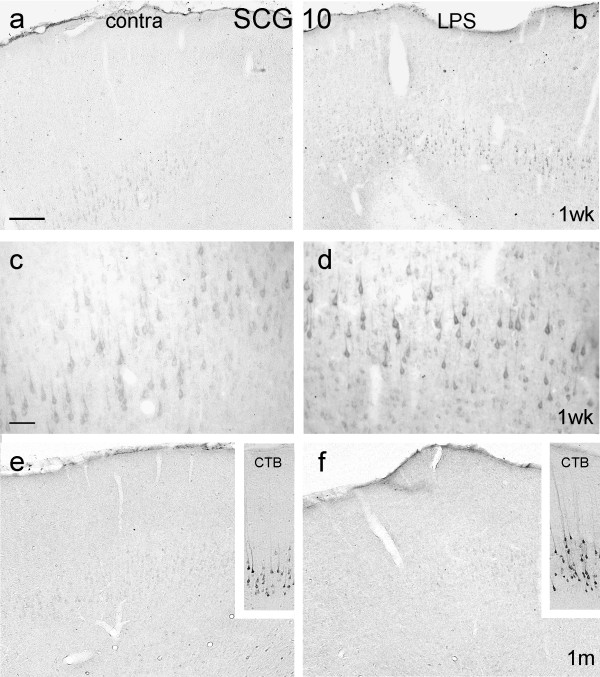
**Expresssion of SCG10 after LPS application**. Coronal sections of motor cortex immunoreacted for SCG10 (except for insets), 1 week (Figs 7a – d) and 1 month (Figs 7e, f) after LPS application. Control, contralateral cortex is shown in Figs 7a, c and e, LPS-treated cortex in Figs 7b, d and f. Note increased SCG10 immunoreactivity in layer V cells at one week in Fig. 7b compared to contralateral cortex (Fig. 7a), and absence of immunoreactivity in more superficial cortex. Fig. 7c is enlarged from layer V in Fig. 7a and Fig. 7d is enlarged from layer V in Fig. 7b. One month after LPS application there is only a background level of SCG10 immunoreactivity in layer V cells of both contralateral (Fig. 7e) and ipsilateral (Fig. 7f) cortex. The insets are from immediately serial sections to Figs 7e and 7f and show retrogradely CTB-labelled CST neurons in layer V. Bar in Fig. 7a = 200 μm and also applies to Figs 7b, e, f and insets; bar in Fig. 7c = 50 μm and also applies to Fig. 7d.

**Figure 8:**
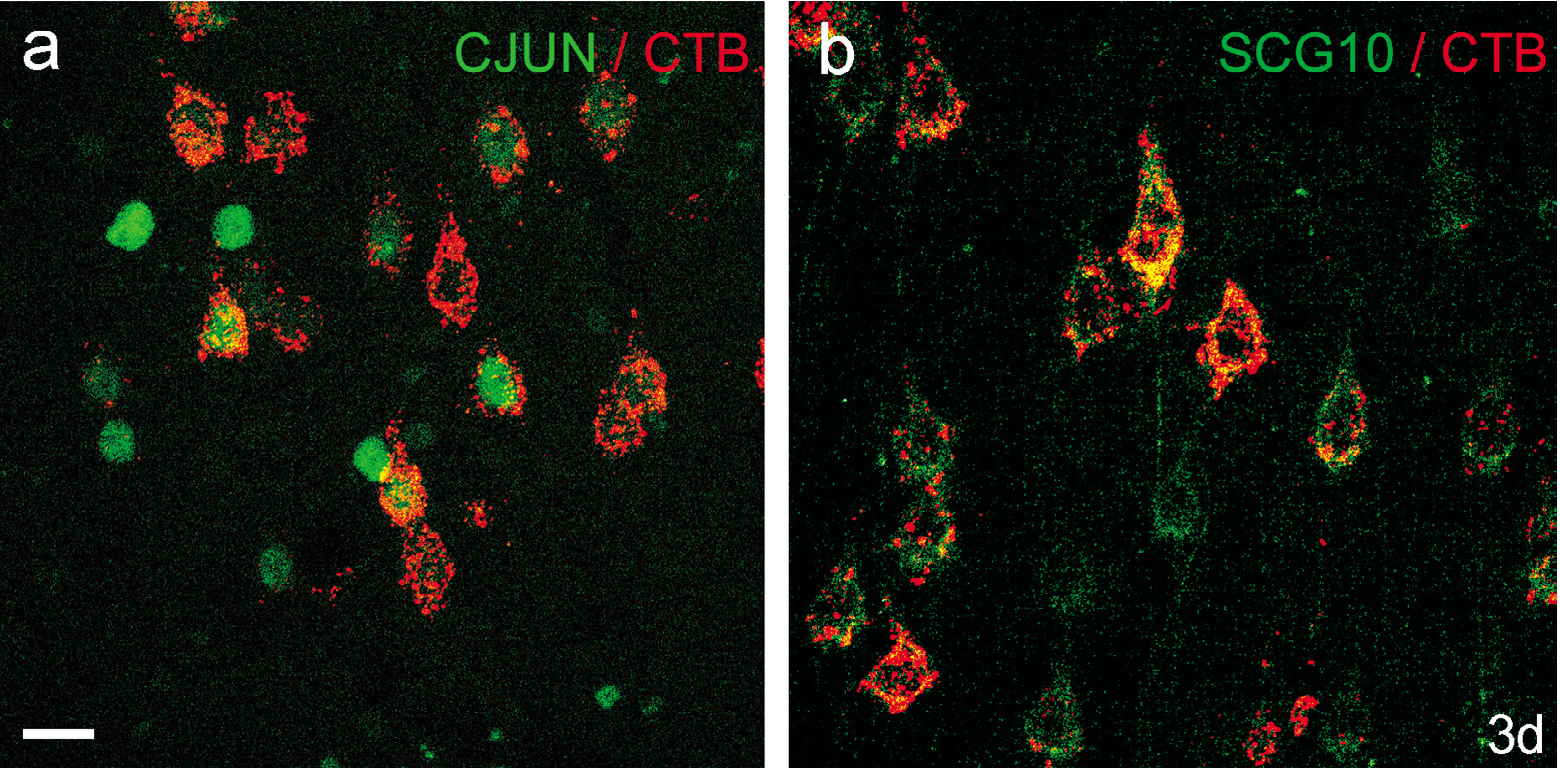
**Co-localisation of retrograde label and c-Jun or SCG10**. CTB (red) in the cell bodies of CST neurons co-localised with c-Jun (Fig. 8a) or SCG10 (Fig. 8b) (green) in coronal sections of the motor cortex (layer V), 3 days after application of LPS and simultaneous injection of CTB into the CST at C4. Note that not all layer V neurons expressing c-Jun in their nuclei also show co-localisation with CTB (Fig. 8a). There is a higher degree of co-localisation between SCG10 and CTB (Fig. 8b). Confocal microscopy; scale bar = 20 μm and applies to both images.

SCG10 was weakly upregulated by layer V pyramidal neurons and by no other cells. This effect was seen from 24 hours to 2 weeks after LPS application (Fig. 7). The cell bodies displaying upregulation of SCG10 were identified as CST neurons (Fig. 8b).

##### GAP-43 mRNA

**Figure 9:**
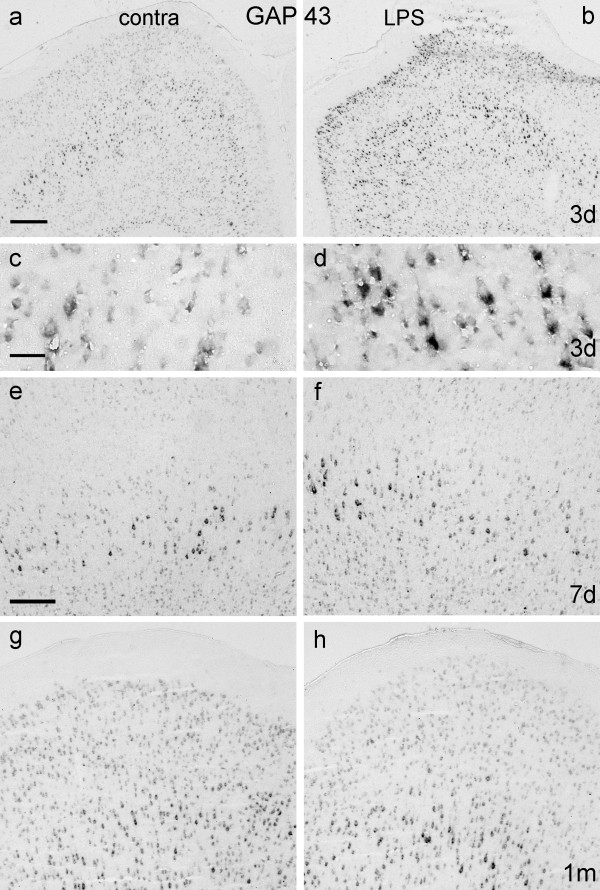
**GAP-43 mRNA expression after LPS application**. Coronal sections of motor cortex hybridised with GAP-43 mRNA probe 3 days (Figs 9a – d), 7 days (Figs 9e, f) and 1 month (Figs 9g, h) after unilateral application of LPS (Figs 9b, d, f, h) or sham operations to the contralateral (control) side (Figs 9a, c, e, g). Background levels of GAP-43 mRNA are seen in contralateral (control) cortex at 3 days but stronger expression is apparent in layers II–V of LPS-treated cortex. Areas of layer V in Fig. 9a and b are enlarged in Fig. 9c and d to better show differences in hybridisation signals. By 7 days (Figs 9e and 9f), GAP-43 mRNA expression appears to be identical on both sides, and remains so one month after LPS application (Figs 9g and h). Scale bar in Fig. 9a = 500 μm and also applies to Fig. 9b; scale bar in Fig. 9c = 50 μm and also applies to Fig. 9d; scale bar in Fig. 9e = 200 μm and also applies to Figs 9f – h.

GAP-43 mRNA was upregulated in neurons in layers II – VI of LPS-treated cortex, 3 days after application (Fig. 9b), most conspicuously in layer V. After 7 days (Figs 9e, f), 2 weeks (not illustrated) or 1 month (Fig. 9g, h) GAP-43 expression resembled that in unoperated cortex.

### Effect of CST injury in cervical spinal cord

Transection of the CST at C4 contralateral to LPS application had no detectable effect on the expression of c-Jun, ATF3 or SCG-10 (not illustrated). Because of these negative findings we did not investigate possible effects of combining LPS treatment with C4 CST injury in the animals used to study GAP-43 mRNA expression by *in situ *hybridisation.

### Sprouting of corticospinal tract axons at the lesion site

**Figure 10:**
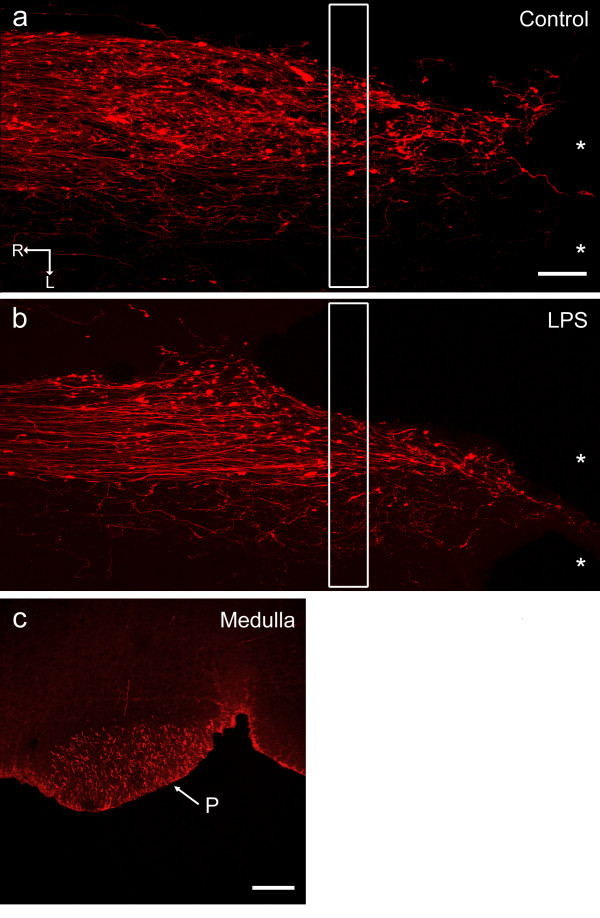
**Anterograde labelling of CST axons after spinal cord injury and LPS application**. Horizontal sections through spinal cord injury sites (Figs 10a, b), and a transverse section through the medulla (Fig. 10c) 21 days after lesion of the CST at C4 with either simultaneous injection of BDA into contralateral motor cortex (Control) or injection of BDA into and application of LPS onto motor cortex (LPS). In both the LPS-treated (Fig. 10b) and control tissue (Fig. 10a), end bulbs are seen at the tips of large numbers of axons. There is little sign of axon branching into the contralateral CST, and the labelled axons extending into the lesion site are located in a strand of spared tissue that extends no more than 50 μm. No axons appear to circumnavigate or regenerate beyond the lesion site. The white boxes correspond to the counting frame windows. Fig. 10c shows BDA-labelled CST axons in the pyramid of the medulla (midline at arrow). All images are confocal; * = lesion site; R = rostral; L = lateral; P = pyramid; scale bar in Fig. 10a = 100 μm and also applies to Fig. 10b; bar in Fig. 10c = 200 μm.

Application of LPS to motor cortex had no obvious effect on the numbers and morphology of microglia around the lesion site in the spinal cord, compared with controls (data not shown). Three weeks after spinal cord lesion, injured CST axons, anterogradely labelled with BDA, terminated proximal to the lesion site, many with swollen axonal tips, in both LPS-treated and control animals (Figs 10a, b). No axons were seen to grow into or distal to the lesion cavity or to bypass the lesion site, or send branches around it. Thus, application of LPS to the cortex appeared to produce no enhancement of axon regeneration into or around the lesion site. There was no statistically significant difference between control and LPS-treated animals in either the 'total sprouting ratios' (t-test, p = 0.12) or the 'lesion site sprouting ratios' (t-test, p = 0.32). These data, summarised in Table 3, suggest that application of LPS to motor cortex has no effect on the sprouting response or regeneration of CST axons across a lesion. The application of LPS to cortex did not cause a reduction of labelled CST axons in the medulla (Table 3; Fig. 10c). This result demonstrates that the uptake and anterograde transport of BDA by CST neurons was unaffected by LPS-induced inflammation.

**Table 3:** Quantification of CST axons

	Lesion site axon total	Branching points	Medulla axon total	Total sprouting ratio	Lesion site sprouting ratio
LPS 1	133	52	466	0.11	0.39
LPS 2	123	100	788	0.13	0.81
LPS 3	146	54	685	0.08	0.36
LPS 4	301	128	519	0.25	0.42
LPS group mean ± SEM			614.5 ± 74.5	0.14 ± 0.04	0.5 ± 0.1
					
Con 1	150	162	998	0.16	1.08
Con 2	273	162	602	0.27	0.59
Con 3	200	78	323	0.24	0.39
Con 4	138	98	439	0.22	0.71
Control group mean ± SEM			590.5 ± 147.5	0.22 ± 0.02	0.69 ± 0.15
Student's t-test p value for LPS-treated vs. control animals			0.89	0.12	0.32

*Notes*: BDA-labelled axons (and branch points) in 4 LPS-treated animals and 4 control animals (LPS not applied) were counted at two sites: 1) in 9 horizontal sections through the cervical spinal cord 0.4 mm rostral to the lesion site, in a 60 μm wide counting frame (labelled axons and branch points); 2) in transverse sections through the medulla (number of labelled axons in pyramid). Total sprouting ratio is the ratio between the number of branch points and the total number of labelled CST axons at the level of the medulla. Lesion site sprouting ratio is the ratio between branch points and labelled axons at the same level (0.4 mm rostral to the lesion site); SEM = standard error of the mean

## Discussion

The inability of axotomised CST neurons to upregulate growth-associated gene expression is believed to be one cause of their failure to regenerate axons [10,11]. We have shown here that application of LPS onto the pial surface of motor cortex produced inflammation – demonstrated by activation of microglia – in most cases throughout the entire depth of cortex and also increased expression of growth-associated genes in some CST neurons. ATF3 expression was upregulated for 1 day (but in very few cells), GAP-43 for 3 days, c-Jun for 2 weeks and SCG10 for 2 weeks in neurons within the inflamed cortex. However, in animals treated with LPS and having a concomitant C4 CST injury there was neither an obvious increase in CST axon sprouting nor any regeneration across or around the lesion site.

### Does inflammation around neuronal perikarya stimulate axonal regeneration?

Two experimental models provide evidence that inflammation around neuronal perikarya can stimulate axonal regeneration in vivo. Injection of Corynebacterium extracts into the DRG both enhances the ability of the central axons of DRG cells to regenerate after injury [1] and stimulates upregulation of GAP-43 and c-jun in their cell bodies [12], thus mimicking some of the effects of a conditioning peripheral nerve lesion. Similarly, Leon et al. [2] have shown that zymosan, a powerful inflammatory agent, injected into the vitreous body of the eye in adult rats, produces inflammation in the retina, upregulation of GAP-43 expression by RGCs and enhanced regeneration of RGC axons following optic nerve injury. The interpretation of these experiments has been questioned by Fischer et al. [13] who suggested that zymosan may injure the lens, releasing factors that stimulate axonal regeneration. However, it has been shown recently that activated macrophages secrete a protein that, in conjunction with cAMP, acts as a powerful promoter of neurite outgrowth [3]. We have shown that LPS-induced inflammation also stimulates neuronal expression of growth-associated genes in the cerebral cortex. However, we did not find enhancement of CST axonal regeneration, perhaps because of an insufficiently strong inflammatory response in layer V, or possibly because of local inhibitors at the injury site.

### LPS-induced cerebral inflammation

LPS, an endotoxin derived from the cell wall of E. coli, is a potent inflammatory agent [14]. It has been extensively used in previous experimental studies in the central nervous system (CNS) and other tissues [15]. The Toll-like receptor 4 (TLR-4) acts as an LPS receptor on microglia [16,17], but is absent from cortical neurons [18]. Although the effects of applying LPS to the surface of the cerebral cortex have not been studied previously, it has been shown that injection of LPS into the cortex results in recruitment of macrophages, activation of local microglia and, later, activation of astrocytes [19,20]. Neutrophils however are not recruited, in contrast to what occurs after LPS injection into peripheral tissues [15], which may explain the absence of overt damage to the brain. LPS applied to the cortical surface in this study markedly increased the number of rounded OX42-positive cells at the surface of the brain and in the meninges (which were presumably peripheral macrophages – see Fig. 2), and activated microglia, increasing their number throughout the motor cortex (Table 1). In most animals, there was a clear gradient of activation from superficial to deep, suggesting that LPS activated microglia in a concentration-dependent manner. LPS can also attract circulating monocytes into the brain but it is not clear to what extent circulating monocytes entered the cortex in our experiments, as there are no reliable cell markers that distinguish macrophages from microglia. Furthermore, the astrogliosis shown by GFAP immunofluorescence in the cortex to which LPS was applied confirms the inflammatory effect of the LPS.

Zymosan has been used to produce inflammation in the retina and promote regeneration in crushed optic nerve. It may be significant that LPS injection into the vitreous body was not a sufficient stimulus to induce regeneration of optic axons [2]. However, the eyeball is a highly 'immune-privileged' site, and more resistant to inflammation than cerebral cortex. Zymosan particle injection into cortex caused substantial cavity formation and a glial scar [21], whereas single injections of LPS into cortex caused minimal cavitation [19,22]. Chronic infusion of LPS into hippocampus produced a focal necrotic lesion at the infusion site, with a surrounding region showing activation of astrocytes and macrophages [23]. Our aim was to cause no damage to the CST cell bodies or their axons within the cortex; we therefore applied LPS to the surface of the cortex. There was no sign of necrosis and it is extremely unlikely that LPS applied to the pial surface would have caused axonal injury to CST neurons. If LPS application had resulted in axonal injury, there would have been reductions in the numbers of BDA-labelled axons in the medulla. The counts of labelled CST axons in LPS-treated versus control animals (no significant differences: Table 3) show that direct axonal injury did not occur. Furthermore, CST neurons showing upregulation of SCG10 and c-Jun were retrogradely labelled from the spinal cord after LPS application, demonstrating that their axons were intact (Fig. 8).

### Role of perineuronal macrophages and microglia in axonal regeneration

Macrophages enter DRGs after a peripheral conditioning lesion and may play a role in the survival or regeneration of axotomised neurons [24-26]. Microglial activation also occurs in the CNS around facial, hypoglossal and spinal motor neurons after axotomy [27,28], probably because of the release of macrophage-colony stimulating factor by the injured neurons [29]. In contrast to regenerating facial nucleus neurons, non-regenerating intrinsic CNS neurons do not attract activated microglia after axotomy [30-32]. However, most evidence suggests that the accumulation of microglia around axotomised neurons is unnecessary for axonal regeneration [33,34] and it has been suggested that the main role of microglia around axotomised neurons is in immune surveillance [35,36]. Nonetheless, even if perineuronal macrophages or microglia are not essential for the regeneration of motor axons, the data from optic nerve regeneration experiments suggests that they may be capable of enhancing the cell body response and thus of promoting regeneration in less regeneration-competent neurons.

### Growth-associated genes, axonal regeneration and perineuronal inflammation

The transcription factor c-Jun has been implicated in both neuronal cell death and survival after injury [37-39] and is consistently upregulated after axotomy in regenerating neurons [40-42]. Recently it has been shown that in mice lacking neuronal c-Jun, both cell death and axonal regeneration after axotomy are reduced [42]. ATF3 is also a transcription factor that is induced in many cell types by a range of stresses [43]. ATF3 has been shown to prevent cell death and to be a neurite growth-promoting factor for cultured neurons, apparently acting through HSP27 [44]. Co-localisation studies indicate that ATF3 and c-jun mRNA are co-expressed in several types of neuron after injury. Transfecting ATF3 alone into two neuron-like cell lines did not cause neurite outgrowth; c-Jun expression alone caused some outgrowth, but co-expression of both ATF3 and c-Jun greatly increased neurite outgrowth [45].

It has been hypothesised that GAP-43 and SCG10 are important for axonal elongation, through their role in cytoskeletal dynamics [46-48]. Motor and DRG neurons upregulate these molecules soon after axotomy. Expression decreases markedly following target reinnervation, but remains high if reinnervation is prevented, suggesting that contact with target tissues regulates expression of these molecules.

The effects of ATF3, GAP-43 and SCG10 individually on axonal regeneration in vivo are difficult to assess. No axonal regeneration experiments have been reported on ATF3 knockout mice [49]. GAP-43 deficient mice show neuroanatomical abnormalities but their CNS appear to be grossly normal and with normal axon growth rates [50]; hence GAP-43 is unlikely to be essential for axonal growth but probably is required for successful axonal pathfinding. Transgenic overexpression of individual growth-associated molecules has induced some sprouting [51], but has not turned non-regenerating neurons into regeneration-competent cells, even into favourable environments such as implanted segments of peripheral nerve or Schwann cells [52,53]. However, overexpression of GAP-43 and CAP23 together, greatly enhanced the ability of ascending dorsal column axons to regenerate into peripheral nerve grafts placed in the spinal cord without a conditioning peripheral lesion [54]. No SCG10 knockout animals have been described.

The molecules we have studied are only examples of a large range of neuronal molecules that may be required for regeneration of axons. Tetzlaff et al. [55] showed that although cut rubrospinal axons do not regenerate; the injury results in prolonged increased levels of GAP-43 in their cell bodies, from which they concluded that it is their failure to maintain tubulin and actin synthesis that prevents regeneration. Since individual growth-associated genes are unlikely to greatly stimulate axonal regeneration, finding a mechanism whereby a whole cascade of such molecules could be upregulated may be necessary to bring about axonal regeneration in otherwise refractory CNS neurons. It seems clear that further studies are needed into the mechanisms of upregulating transcription factors that control growth associated proteins.

We have not included in this report the results of studies on the effects of applying zymosan to the pial surface of the motor cortex because we found that half of the animals thus treated developed cortical damage, often severe, in layers I to IV, that made it impossible to distinguish the effect of inflammation from other destructive processes (unpublished findings). Interestingly, the CST neurons in these zymosan-treated rats displayed expression patterns of growth-associated proteins that were almost identical to those seen in LPS-treated rats at corresponding time points. As zymosan is more potent an inflammmogen than LPS, it is possible that LPS stimulated a maximal expression of growth-associated proteins in CST cell bodies without causing overt damage. Furthermore, the estimated number of cells that upregulated c-Jun, SCG10 and GAP-43 after LPS application was far greater than the number of BDA-labelled fibres in our spinal cord injury experiments, suggesting that the cell bodies of these labelled axons had probably upregulated growth-associated genes.

An intracortical injection of a solution of LPS might induce a stronger inflammation closer to the cell bodies of CST neurons, but such an approach is problematic because direct tissue damage may elicit responses which mask/confound those due to the induction of an inflammatory response by cortical application of LPS. We carried out experiments in which small amounts of LPS were injected into deep cortex. Such injections resulted in neither a detectably greater inflammatory response nor greater upregulation of growth-associated proteins in layer V (unpublished results). It remains to be seen whether other approaches to inducing cortical inflammation would promote a stronger and longer-lasting expression of growth-associated proteins, with the effect of stimulating CST axon regeneration.

It is possible that CST axotomy interrupts target derived regeneration-suppressing signals, and this may potentiate the effects of LPS in promoting growth-associated gene expression. However, our results showed no difference in growth associated protein expression between axotomised and intact CST neurons. Previous work in this laboratory has demonstrated that cervical axotomy is insufficient to induce upregulation of growth associated genes, although axotomy within the cortex (close to the cell body) does so [8]. However, promoting enhanced cell body responses by proximal axotomy is unlikely to be of practical value because of the very large distance over which the CST axons would have to regrow to effect functional reconnection.

It is noteworthy that the inflammatory response induced by LPS resulted in more extensive upregulation of ATF3 in glia than in neurons. It is interesting to compare this finding with observations on ATF3 expression in glial cells during Wallerian degeneration. Following dorsal root injury, ATF3 is strongly upregulated in Schwann cells around degenerating axons in the dorsal root, but not in the CNS glial cells around the degenerating axons in the dorsal column [56].

## Conclusion

We carried out these experiments to test the hypothesis that inflammation would induce the expression of growth-associated genes in the cell bodies of CST neurons and would consequently increase the regenerative response after CST injury. We found that application of LPS to the motor cortex induced upregulation of c-jun, ATF3, SCG10 and GAP-43 in some neurons. However, the response was generally greatest in superficial layers, as was the degree of inflammation. Nonetheless, we have demonstrated that perineuronal inflammation produced by a single application of LPS can cause CST neurons to upregulate a range of growth-associated proteins, although the number of cells which responded in this way was limited and the effects most obvious in the first few days after application. Axonal regeneration was not enhanced. The failure of CST neurons to show enhanced axon regeneration may be due to the inflammatory stimulus not being strong enough or not lasting long enough. There was variation between animals in the extent of inflammation/microglial activation produced by the standard dose (500 μg) of LPS applied to the cortical surface, and inflammation was always less marked in layer V than near the surface of the cortex. The absence of a widespread upregulation of ATF3 in CST neurons may have been the result of insufficient inflammation in layer V, which may have limited the expression of the full repertoire of downstream genes necessary for regeneration. Sustained, or increased inflammation deeper in the cortex may be necessary to induce a sufficiently strong and prolonged upregulation of growth-associated genes that would lead to a vigorous regenerative response to injury by CST neurons.

## Methods

### Animals and surgery

Adult female Sprague-Dawley rats (220–250 g; n = 66) were anaesthetised with a mixture of halothane, nitrous oxide and oxygen. All surgery was performed under aseptic technique and was approved by the local ethical committee and by the Home Office under UK animal experimental legislation.

### Application of lipopolysaccharide

The scalp was incised in the midline and bilateral craniotomies performed to expose the surface of the motor cortex (localised according to the atlas of Paxinos and Watson, [57]). A 3 mm × 4 mm piece of parietal bone was removed from the cranium, centred 3 mm caudal to the bregma and 2 mm from the midline. The dura was opened and 500 μg of LPS from *Escherichia coli*, serotype 055:B5 (Sigma, UK) was applied – as a powder – to the exposed pial surface of the right hemisphere (Fig. 1a). The powder dissolved to a paste on contact with the CSF and was kept in place by application of Gelfoam (Johnson & Johnson, Skipton, UK), which was anchored to the cranium with histoacryl glue (B. Braun, Melsungen AG, Germany). In preliminary experiments it was found that if less than 500 μg of LPS was applied to cortex, inflammation did not consistently extend as deep as layer V. As a control for the effects of surgery, the contralateral cortex was exposed but LPS was not applied. The scalp incision was closed with Michel clips and the animals allowed to recover in an incubator. Survival times were 1 day (n = 3), 3 days (n = 8), 7 days (n = 6), 14 days (n = 4) and 1 month (n = 4) after LPS application. A further 9 LPS-treated animals (survival 3 days to 1 month) also received a left corticospinal tract (CST) transection lesion at the time of LPS application in order to determine if concomitant axotomy is a requirement for growth associated gene expression. A C3/4 laminectomy was performed and microscissors used to cut the area between the left dorsal root entry zone and the right CST down to a depth of 2 mm. The dura was closed and the wound closed in layers, and the animals allowed to recover in an incubator. In 6 other animals (survival 3, 7 and 14 days), the motor cortex was exposed unilaterally, Gelfoam applied on top of the burrhole and the scalp then closed (without the application of LPS). In 3 further animals (survival 3 days) the motor cortex was exposed but neither LPS nor Gelfoam were applied prior to closing the scalp, as a control for the possibility that the surgical procedure or the application of Gelfoam contributed to any of the effects observed (see Table 4).

**Table 4:** Animal utilisation

Survival time	Cortical LPS (with SCI) for IHC	Cortical LPS with retrograde labelling	Cortical LPS for ISH	Cortical LPS with SCI and anterograde labelling	SCI with anterograde labelling	Controls (Gelfoam not applied)
1 day	3					
3 days	5 (+3)	5	4 (+1 control)			3 (+3)
7 days	4 (+2)		4			1
14 days	2 (+2)	2	3			2
21 days				4	4	
1 month	2 (+2)	1	4			
TOTAL	16 (+9) = 25	8	15 (+1)	4	4	6 (+3)

*Notes*: IHC = immunohistochemistry; ISH = in situ hybridisation; SCI = spinal cord injury (denotes animals with unilateral transection of corticospinal tract at C4).

Controls were animals in which the dura was opened unilaterally without application of LPS (in 6 of these animals Gelfoam was applied to the cortex; in 3 others no Gelfoam was applied). In addition, control exposure of the left cerebral cortex was carried out in all animals with unilateral LPS application.

This Table does not include 6 positive control animals in which the expression of growth associated proteins was examined in the facial nucleus following facial nerve injury and it also excludes 12 animals used in pilot studies to determine the optimal dose of LPS.

### Retrograde tracing with Cholera Toxin B (CTB) or Fluorogold (FG)

In order to identify the neuronal cell bodies giving rise to the CST in animals treated with LPS, the contralateral cervical spinal cord was injected with CTB (List laboratories, Inc, Campbell, CA, USA) in a further 5 rats. Three days before the animals were killed, a laminectomy was performed at C3/4, the dura opened, and 1.5 μl of CTB was slowly injected into the CST, through a Hamilton syringe attached to a fine glass pipette (Fig. 1b). The dura and wound were closed in layers and the animals allowed to recover in an incubator. The survival periods after LPS application for these animals were 3 days (n = 2), 14 days (n = 2) and one month (n = 1). Coronal sections of brain were processed for the visualisation of both growth associated proteins and CTB (described below). In a further 3 animals (survival 3 days) Gelfoam impregnated with 1.5 μl of Fluorogold (Molecular Probes, Oregon, USA) (diluted to 2% in sterile water) was inserted into a contralateral (left) C6 corticospinal tract lesion at the same time as LPS was applied to the cortex. Coronal sections of the brains of these three animals were immunostained with OX42 antibody in order to examine the relationships between microglia and CST cell bodies after LPS treatment (see below).

### Corticospinal tract injury and anterograde tracing with Biotinylated Dextran Amine (BDA)

To study the effect of cell body inflammation on CST axon regeneration, the CST was lesioned at the same time as LPS was applied to the cerebral cortex (n = 4) or the dura opened without application of LPS (n = 4), and at the same time BDA was injected into the cortex to label CST axons (Fig. 1c). The spinal cord was exposed at C3/4 by laminectomy and the area between the left dorsal root entry zone and the midline was cut to a depth of 2 mm with microscissors, and the cut extended to cross the midline, undercutting the midline spinal artery to minimise ischaemic damage. Overlying muscle and skin was sutured. Immediately thereafter, a midline incision was made over the skull, a 4 mm × 2 mm burr hole made over the right parietal cortex, the dura opened and LPS powder (500 μg) placed on the pial surface (LPS application omitted in control animals). Then BDA (Molecular Probes, Oregon, USA) dissolved in 0.1 M phosphate-buffered saline (PBS) (10% solution) was injected into the cortex, about 1 mm below the pial surface, through a Hamilton syringe attached to a glass micropipette. The micropipette was introduced into the cortex at a very shallow angle from the rostral edge of the burr hole and advanced 4 mm in a caudal direction, and parallel to the cortical surface. The BDA solution (2.5 μl) was injected as the pipette was slowly withdrawn to its entry point. The injection was then repeated, via the same entry point, but with the micropipette directed 1 mm medial to the initial injection track, and repeated again, with the pipette directed 1 mm lateral, thus delivering a total of 7.5 μl BDA solution. The exposed area of cortex was covered with Gelfoam, the wound sutured and the animal checked upon recovery to ensure that no unexpected deficits were present. Survival time for all animals in this group was three weeks.

Any inflammatory response due to damage induced by the relatively large volumes of BDA injected would occur in both LPS-treated and control animals and would therefore not significantly impact on or obscure effects due to LPS.

### Facial nerve injury

As a positive control, expression of growth-associated proteins was induced in facial nucleus neurons by facial nerve injury. In 6 adult rats, anaesthetised as described above, a 1 cm skin incision was made posterior to the right ear, and the facial nerve exposed and crushed for 10 seconds with watchmakers' forceps proximal to its branch point. The skin incision was closed with Michel clips and the animals allowed to recover in an incubator. All were killed after seven days.

### Perfusion and histological processing

Animals were overdosed with halothane and intraperitoneal pentobarbitone, and perfused transcardially with 200 ml of PBS followed by 350 ml of 4% paraformaldehyde in 0.1 M PBS buffer. The brain was removed and immersed in fixative solution for 2 hours. Dissected tissue specimens were then cryoprotected for 40 hours in PBS containing 30% sucrose.

### Antibodies

#### ATF3

polyclonal (raised in rabbit); dilution 1:800; source – Santa Cruz, CA, USA.

#### c-Jun

polyclonal (raised in rabbit); dilution 1:5000; source – Dr. A. Behrens (Cancer Research, UK), gift.

#### SCG10

polyclonal (raised in rabbit); dilution 1:3000; source – Dr. G. Grenningloh (University of Lausanne, Switzerland), gift.

#### OX42

monoclonal (raised in mouse); dilution 1:3000; source – Serotec, Oxford, UK.

#### GFAP

monoclonal (raised in mouse); dilution 1:800; source – Sigma, St. Louis, Missouri, USA

#### CTB

polyclonal (raised in goat); dilution 1:100,000 – List Biological Laboratories, CA, USA.

### Single label immunohistochemistry

Coronal sections of brain through the motor cortex and pons (containing the facial nucleus) were cut at 40 μm using a freezing microtome and collected in 0.1 M PBS. Care was taken to ensure that every section to be reacted for growth-associated protein expression was immediately adjacent to a section reacted for CTB. All sections were then washed in 0.3% H_2_O_2 _for 15 minutes to remove endogenous peroxidase, followed by 3 × 5 minute PBS washes (or 0.1 M tris-buffered saline (TBS) + 0.05% Tween 20 (TNT), for OX42 and CTB). Then followed a one hour wash in blocking solution, using 1% bovine serum albumin (BSA), 0.1% Triton-X and 10% normal goat serum in PBS (for sections to be reacted with the antibodies against ATF3, c-Jun and SCG10) or 2% horse serum in TBS (for OX42, GFAP and CTB). Serial sections were incubated with primary antibody (made up in appropriate blocking solution) against ATF3, c-Jun SCG10, OX42, GFAP or CTB for 72 hours at 4°C. Sections of motor cortex and pons incubated with the appropriate blocking solution rather than with primary antibody, served as negative controls. After 3 washes in PBS/TNT, sections to be immunoreacted for ATF3, c-Jun and SCG10 were incubated for 2 hours in 1:200 rat-adsorbed, goat anti-rabbit biotinylated secondary antibody; sections to be immunoreacted for CTB were incubated in 1:200 horse anti-goat biotinylated secondary antibody and sections to be immunoreacted for OX42 or GFAP were incubated in horse anti-mouse biotinylated secondary antibody (all diluted in appropriate blocking solution). After 3 further washes, ATF3, c-Jun, SCG10 and CTB sections were reacted with an avidin-biotin complex (ABC) kit (Vectastain, Burlingame, CA, USA) for 2 hours, again washed in PBS and finally reacted with 0.04% 3-3' diaminobenzidine tetrahydrochloride in 0.015% H_2_O_2 _in TBS until a brown reaction product appeared (usually about 12–15 minutes). OX42 sections were washed in TNT, incubated in streptavidin-conjugated horseradish peroxidase (streptavidin-HRP) (Vector Laboratories, Burlingame, CA, USA) for 1 hour at 1:200 in TBS, washed in TNT buffer, reacted with Tyramide Cy3 (NEN Life Science Products, Boston, USA) for 30 minutes at 1:400 in TBS and then washed in TBS. GFAP sections were washed in TNT, incubated in horse anti-mouse IgG FITC conjugate (Sigma, St. Louis, Missouri, USA) at 1:100 for 1 hour and then washed in TBS. Sections for fluorescence microscopy were mounted onto agar-coated slides and coverslipped immediately with glycerol containing 1,4-diazabicyclo[2,2,2]octane (DABCO). Sections reacted with ABC were dried overnight and dehydrated through ascending concentrations of alcohol, followed by Histoclear (National Diagnostics, Georgia, USA) before being mounted onto slides and coverslipped with DPX (Merck, Poole, UK).

### Double label immunohistochemistry

The steps followed were the same as for OX42 immunofluorescence processing until the stage of incubation in primary antibody solution. At this point, primary antibody against ATF3, c-Jun or SCG10 was mixed with the antibody against CTB, made up in blocking solution containing 2% normal horse serum. Sections were then rinsed in TNT (3 × 5 minutes), incubated for 2 hours in 1:200 horse anti-goat biotinylated secondary antibody (made up in TNT), rinsed again in TNT and incubated for 1 hour in a mixture of 1:200 streptavidin-HRP and 1:100 donkey anti-rabbit FITC (made up in blocking solution). After 3 further TNT washes, sections were reacted for 30 minutes in 1:400 Tyramide Cy 3, washed in TBS, mounted onto slides and coverslipped immediately with DABCO.

The steps followed for retrograde labelling with Fluorogold were the same as for single OX42 immunofluorescence processing up to the stage of incubation with the ABC kit. At this point, sections were instead incubated with 1:200 horse anti-mouse Alexa Fluor 568 (Molecular Probes, Invitrogen Corporation, Paisley, UK) for one hour, washed in TBS, mounted onto slides and coverslipped immediately with DABCO.

Digital images were taken using a Zeiss Axiophot microscope equipped with Openlab image processing software.

### BDA labelling immunohistochemistry

Cryoprotected spinal cord or brainstem tissue was frozen in Tissue-Tek (Sakura, Zoeterwoude, Netherlands) cooled with dry ice. Serial horizontal cryostat sections through the entire CST and transverse sections of the medulla through the pyramids, were cut at 40 μm and collected in 0.05 M TBS. The free floating sections were rinsed in 0.05 M TBS and 0.5% Triton X-100 (TBST), incubated in 0.3% H_2_O_2 _for 15 minutes, rinsed in TBST (2 × 10 minutes) and incubated at 1:200 dilution of ABC overnight at 4°C. They were then rinsed in TBST, incubated in Tyramide Cy3 at 1:400 for 30 minutes, rinsed in 0.05 M TBS and mounted as above.

### In situ hybridisation

An additional 16 animals were used for in situ hybridisation studies. Of these, 15 had unilateral LPS application to the pial surface of motor cortex as described above and 1 had a unilateral opening of dura only (as a control). They were sacrificed at 3 days (n = 4, plus 1 control), 7 days (n = 4), 14 days (n = 3) and 1 month (n = 4) after LPS application, by lethal overdose of halothane and intraperitoneal pentobarbitone. Their brains were removed and immediately frozen in Tissue-tek cooled with dry ice.

GAP-43 cRNA antisense probes were obtained from pcDNA-GAP-43, which contains the 680 base pair open reading frame of rat GAP-43 cDNA and labelled with digoxigenin according to the manufacturer's recommendations using an RNA labelling kit (Boehringer Mannheim, Germany), as described by Mason *et al*. [58]. Sections derived from animals at all survival times were processed under identical conditions, at the same time.

In situ hybridisation was carried out as previously described [59,60]. Coronal cryostat sections of motor cortex directly under the area of LPS application were cut at a nominal thickness of 12 μm, thaw-mounted onto slides coated with 3-aminopropyltriethoxy-silane, and fixed with 4% paraformaldehyde in PBS overnight at 4°C. After washing in PBS, sections were treated with 0.1 M HCl, and washed in PBS, incubated in 0.1 M triethanolamine containing 0.25% acetic anhydride, and then washed with PBS, dehydrated in an ascending ethanol series, and air dried. Prehybridisation was carried out at 37°C for 3 hours with a mixture of prehybridisation buffer/deionised formamide 1:1 (containing 50% formamide, 25 mM ethylenediaminetetra-acetic acid (EDTA), 50 mM, pH 7.6 Tris-HCl, 2.5× Denhardt's solution, 0.25 mg/ml tRNA (Boehringer Mannheim), and 20 mM NaCl). The digoxigenin-labelled GAP-43 probe was prepared at a concentration of 3 μl/ml with hybridisation buffer containing 50% formamide, 20 mM Tris-HCl (pH 7.50), 1 mM EDTA, 1× Denhardt's solution, 0.5 mg/ml tRNA, 0.1 mg/ml poly (A) RNA (Sigma), 0.1 M DTT, and 10% dextran sulfate. Hybridisation was performed overnight at 62°C. After hybridisation, sections were washed in 0.2× standard saline citrate (SSC), containing 30 mM NaCl, and 3 mM Na-citrate, pH 7.0 and then in 0.1× SSC/50% formamide at the hybridisation temperature. Sections were equilibrated with buffer 1 (100 mM Tris-HCl, 150 mM NaCl, pH 7.5), incubated in modified buffer 2 (1% Boehringer blocking reagent, 0.5% BSA fraction from Sigma in buffer 1) and then incubated with alkaline phosphatase-coupled antibodies to digoxigenin (Boehringer Mannheim, Germany) at a dilution of 1:700 in modified buffer 2 overnight at 4°C. Sections were washed in buffer 1, equilibrated in buffer 3 (100 mM Tris-base, 100 mM NaCl, 50 mM MgCl_2_, adjusted to pH 9.5), and developed in the dark with buffer 3 containing 0.34 mg/ml 4-nitroblue tetrazolium chloride (Sigma), 0.175 mg/ml 5-bromo-4-chloro-3-indolylphosphate (Sigma), and 0.25 mg/ml levamisol (Sigma). Development was stopped by washing with buffer 4 (10 mM Tris-HCl, 1 mM EDTA, pH 8.0), following which the sections were dried and mounted in DPX beneath glass coverslips.

### Quantification of OX42-labelled microglia

In three LPS-treated animals surviving 3 days, 14 days and 1 month after application of LPS, microglia were counted in layer V of cortex under the area of LPS application and under the area of the contralateral (control) burrhole. For each animal, activated and ramified microglia were counted within a counting frame of 400 μm × 340 μm superimposed on a digital image of layer V captured using a ×20 objective lens. The number of activated and ramified microglia (means of 3 counts) was calculated as well as the relative proportions of activated and ramified microglia (expressed as percentages) for each survival time (see Table 1).

### Quantification of anterogradely labelled corticospinal tract axons

In the four LPS-treated animals in which BDA was injected into motor cortex, and the four control animals (BDA injected; no LPS applied), consecutive serial horizontal sections through the entire cervical lesion site (9 sections per animal) and 12 transverse sections through the medulla were cut at a thickness of 40 μm and scanned with a Leica confocal microscope. Projection images used an accumulation of 3 scans of each optical section and represent stacks of 20–25 optical sections merged together, with a resolution of 1024 × 1024 pixels. The source of these digital images was then blinded from the quantifier. Images were standardised to 500 μm × 500 μm. For the horizontal sections, a frame was made which hid each image from view except for a 60 μm wide window, running transversely across the image, orientated perpendicular to the CST (corresponding to the white boxes in Fig. 9). The total number of BDA-labelled axons traversing this window, positioned 0.4 mm rostral to the lesion site, were counted through the 9 serial images of the CST. The number of axons traversing the window and branching within it were separately recorded from the serial horizontal sections and totalled for each animal. The total number of labelled CST axons in each animal was estimated by counting the number of labelled axons in a transverse section of the medullary pyramid, photographed at a magnification of 60× (mean of counts of 3 sections).

Anterograde labelling is variable, presumably because of variations in the uptake of BDA in the motor cortex, and can cause bias if *total *numbers of labelled axons or branch points are compared between animals. In order to normalise for differences in the tracing efficiency in individual animals, the total number of branch points was divided by the total number of labelled CST axons in the pyramid (Fig. 10c) to give a 'total sprouting ratio' for each animal. Another estimate of the proportion of the CST axons sprouting near the lesion was obtained by dividing the total number of branch points 0.4 mm rostral to the lesion site by the total number of axons counted 0.4 mm rostral to the lesion site to give a 'lesion site sprouting ratio' for each animal. After unblinding the results, the two sets of ratios for the 4 LPS-treated animals and the 4 control animals were analysed for significant differences by using a two-tailed unpaired Student's t-test. It should be noted that the number of labelled CST axons counted in the medulla gives a more accurate estimate of the total number of axons labelled with BDA than do counts close to the lesion site. Thus, the 'total sprouting ratio', which utilises the medullary count, is probably a better estimate of the injured CST regenerative response than the 'lesion site sprouting ratio'.

### Control studies

#### Positive controls

c-Jun and ATF3-positive nuclei were identified in the ipsilateral facial nucleus, one week after facial nerve crush. SCG10-positive cells were found in both facial nuclei, but immunoreactivity was greater in the facial nucleus ipsilateral to the crushed facial nerve. These results were consistent for all animals.

#### Negative controls

When the primary antibody was omitted, no cells displayed immunoreactivity for c-Jun, ATF3 or SCG10 in brain or facial nucleus. No positive control tissue was used in the GAP-43 experiments; the contralateral and unoperated cortex was used as a negative control.

## List of Abbreviations Used

ABC Avidin Biotin Complex

BDA Biotinylated Dextran Amine

BSA Bovine serum albumin

C4 4^th ^cervical vertebral level

C6 6^th ^cervical vertebral level

CNS Central nervous system

CSF Cerebrospinal fluid

CST Corticospinal tract

CTB Cholera toxin – Subunit B

DABCO 1,4-diazabicyclo[2,2,2]octane

DRG Dorsal root ganglion

FG Fluorogold

GFAP Glial fibrillary acidic protein

HRP Horseradish peroxidase

LPS Lipopolysaccharide

PBS Phosphate-buffered saline

RGC Retinal ganglion cell

SCI Spinal cord injury

SSC Standard saline citrate

TBS Tris-buffered saline

TBST TBS + Triton X-100 (0.5%)

TNT TBS + Tween 20 (0.05%)

## Authors' contributions

PNA and ARL designed this study. KHI performed the surgery, with advice from PNA and KR. KHI did most of the lab work, with help from JKM for immunohistochemistry and MRJM for in situ hybridisation. The data analysis was done by KHI, who also wrote major parts of the paper, with contributions from PNA and ARL.
